# Landscape of gut mucosal immune cells showed gap of follicular or memory B cells into plasma cells in immunological non‐responders

**DOI:** 10.1002/ctm2.1699

**Published:** 2024-05-23

**Authors:** Zerui Wang, Cheng Zhen, Xiaoyan Guo, Mengmeng Qu, Chao Zhang, Jinwen Song, Xing Fan, Huihuang Huang, Ruonan Xu, Jiyuan Zhang, Jinhong Yuan, Weiguo Hong, Jiaying Li, Fu‐Sheng Wang, Yan‐Mei Jiao, Enqiang Linghu

**Affiliations:** ^1^ Senior Department of Gastroenterology the First Medical Center of Chinese PLA General Hospital Beijing China; ^2^ Senior Department of Infectious Diseases the Fifth Medical Centre of Chinese PLA General Hospital National Clinical Research Center for Infectious Diseases Beijing China

**Keywords:** B cells, gut, HIV, plasma cells, single‐cell RNA sequencing

## Abstract

**Background:**

The gut is an important site for human immunodeficiency virus (HIV) infection and immune responses. The role of gut mucosal immune cells in immune restoration in patients infected with HIV undergoing antiretroviral therapy remains unclear.

**Methods:**

Ileocytes, including 54 475 immune cells, were obtained from colonoscopic biopsies of five HIV‐negative controls, nine immunological responders (IRs), and three immunological non‐responders (INRs) and were analyzed using single‐cell RNA sequencing. Immunohistochemical assays were performed for validation. The 16S rRNA gene was amplified using PCR in faecal samples to analyze faecal microbiota. Flow cytometry was used to analyze CD4+ T‐cell counts and the activation of T cells.

**Results:**

This study presents a global transcriptomic profile of the gut mucosal immune cells in patients infected with HIV. Compared with the IRs, the INRs exhibited a lower proportion of gut plasma cells, especially the IGKC^+^IgA^+^ plasma cell subpopulation. IGKC^+^IgA^+^ plasma cells were negatively associated with enriched *f. Prevotellaceae* the INRs and negatively correlated with the overactivation of T cells, but they were positively correlated with CD4^+^ T‐cell counts. The INRs exhibited a higher proportion of B cells than the IRs. Follicular and memory B cells were significantly higher in the INRs. Reduced potential was observed in the differentiation of follicular or memory B cells into gut plasma cells in INRs. In addition, the receptor‐ligand pairs CD74_MIF and CD74_COPA of memory B/ follicular helper T cells were significantly reduced in the INRs, which may hinder the differentiation of memory and follicular B cells into plasma cells.

**Conclusions:**

Our study shows that plasma cells are dysregulated in INRs and provides an extensive resource for deciphering the immune pathogenesis of HIV in INRs.

**Key points:**

- An investigation was carried out at the single‐cell‐level to analyze gut mucosal immune cells alterations in PLWH after ART.
- B cells were significantly increased and plasma cells were significantly decreased in the INRs compared to the IRs and NCs.
- There are gaps in the transition from gut follicular or memory B cellsinto plasma cells in INRs.

## Graphical abstract

(1) An investigation was carried out at the single‐cell level to analyse gut mucosal immune cell alterations in PLWH after ART. (2) B cells were significantly increased and plasma cells were significantly decreased in the INRs compared to the IRs and NCs. (3) There are gaps in the transition from gut follicular or memory B cells into plasma cells in INRs.

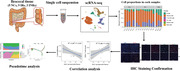

## 1 INTRODUCTION

The gastrointestinal tract (GIT) is a major organ in which early acute human immunodeficiency virus (HIV) infection can result in the extensive depletion of mucosal CD4^+^ T cells, which serve as target cells for the virus.^1^ Notable depletion of the mucosal CD4^+^ T cells, together with epithelial apoptosis induced by HIV infection, disrupts gut epithelial and mucosal barriers, which increases intestinal permeability and leads to further in vivo immune overactivation.^2^ Antiretroviral therapy (ART) can promote the gradual restoration of peripheral CD4^+^ T cells in people living with HIV (PLWH). For example, the immune function of some PLWH is restored after long‐term ART, and they are considered immune responders (IRs).^3^ Conversely, approximately 10%−40% of PLWH fail to normalize their CD4^+^ T cells and are usually considered immune non‐responders (INRs), however, this term, lacks a uniform definition.^4^

Increased risk of AIDS‐related and non‐AIDS‐related diseases occurs in INRs compared with IRs.^5^ Although the exact mechanisms contributing to the immune response failure of patients remain unclear, the time to ART initiation is likely a major factor influencing INRs incidence.^6^, ^7^ Single‐cell RNA sequencing (scRNA‐seq) technologies have been used to investigate the global transcriptional profiles of peripheral immune cells in IRs and INRs and have found that impaired function of MAIT cells is associated with host immune failure in INRs.^8^

Evidence has shown that INRs also exhibit a greater degree of exhaustion of mucosal and blood CD4^+^ T cells than IRs,^1^ while HIV replication in GIT may continue to occur due to incomplete immune restoration in INRs.^9^, ^10^ Our previous study revealed that INRs had higher regulatory T cells (Tregs) and lower Th17 percentages than IRs. Furthermore, the Th17/Treg ratio was negatively correlated with intestinal fatty acid binding protein levels (a marker of intestinal barrier damage).^11^ These results demonstrated that the disruption of the intestinal barrier was associated with gut immune cell alteration in PLWH. As a result of intestinal barrier damage, gut microbial antigens may continuously cross the leaky epithelial barrier and subsequently induce systemic immune inflammation, spatial alteration in T/B‐cell homeostasis, and overactivation of immune cells.^12^ Therefore, understanding how gut mucosal immune cell alteration occurs in PLWH undergoing ART may be of substantial clinical relevance.^10^

Chen et al.^13^ discovered that IgA enrichment in the gastrointestinal mucosa can maintain the physiological homeostasis of symbionts and protect epithelial cells from pathogen attack. However, the relationship between INRs and intestinal IgA production remains unclear. Notably, previous studies have examined the pathological and immune cell characteristics of the gut based only on gut tissue pathology and have not yet disclosed the landscape or interaction of gut immune cells among PLWH undergoing ART. An integrated view of the holistic immune cell profile is still lacking in the gut mucosal tissue of PLWH undergoing ART, including IRs and INRs, which makes it difficult to gain insight into gut immune pathogenesis and its relation to immune restoration and clinical outcomes in these patients. To address these issues, we investigated the global transcriptional profiles of gut mucosal immune cells from the ileocecal mucosal tissue of PLWH receiving ART and HIV‐negative controls. Our findings disclosed a significant reduction in the levels of IgA^+^ plasma cells, especially IGKC^+^IgA^+^ plasma cells in the INRs.

## 2 MATERIALS AND METHODS

### 2.1 Study participants

This study enrolled 12 male individuals with chronic HIV infection who had undergone successful ART (with a viral load remaining undetectable after 6 months of ART) for more than 2.5 years (Table 1). Among them, nine were IRs with a CD4^+^ T‐cell count greater than 500 cells/μL, and the remaining three were INRs with a CD4^+^ T‐cell count less than 200 cells/μL. In addition, five HIV‐negative men who underwent intestinal examinations were enrolled as control participants (NCs). Individuals with a defined intestinal disease, tuberculosis, or a moribund status were excluded. Individuals with a history of smoking, drinking alcohol, or taking probiotics or prebiotics preceding the last month were also excluded.

**TABLE 1:** Characteristics of patients infected with HIV and negative controls enrolled in this study.

	NCs (*n* = 5)	IRs (*n* = 9)	INRs (*n* = 3)
Age (year)	42 (35, 47)	40 (29, 48)	45 (36, 57)
Gender (male/female)	5/0	9/0	3/0
Nadir CD4 count (cells/uL)	–	309 (257, 382)	85 (10, 92)
ART regimens			
2NRTIs+1NNRTIs	–	5	2
2NRTIs+1INSTIs	–	4	1
ART duration (years)	–	4 (3, 5)	3.5 (2.6, 4.5)
Viral load (copies/mL)	–	<LDL	<LDL
CD4 count (cells/uL)	921 (627, 1376)	878 (517, 1428)	133 (132, 142)
CD8 count (cells/uL)	479 (308, 902)	1049 (601, 1559)	482 (475, 712)
CD4/CD8 ratio	1.98 (1.37, 2.41)	.75 (.48, 1.45)	.27(.2, .28)

Abbreviations: INRs, immunological non‐responders; IRs, immune responders; INSTIs, integrase strand transfer inhibitors; “‐”, not applicable; LDL, lower than detectable level; NCs, HIV negative controls; NNRTIs, non‐nucleoside reverse transcriptase inhibitors; NRTIs, nucleoside reverse transcriptase inhibitors.

### 2.2 Gut tissue collection and preparation

Ileocyte mucosal tissue samples were obtained from the enrolled patients using colonoscopy and were quickly immersed in a tissue preservation solution, as previously reported.^14^, ^15^ The tissue samples were kept at 4°C and cut into small pieces within 24 h. After the tissue samples were digested using 1 mg/mL collagenase IV at 37°C for 30 min with shaking, the cells were washed with 1X DPBS containing 2% FBS and sieved through a 40 μm cell strainer after 70 μm cell strainer (BD Biosciences). The red blood cells were suspended in red blood cell lysis buffer (Solarbio) and incubated on ice for 2 min. After two washes, the cells were suspended in 1X DPBS containing 2% FBS. The survival rate of all cells exceeded 85%.

### 2.3 Library preparation and sequencing

Cell suspensions from gut mucosal cells were prepared following the 10× Chromium3′ version v3.1 kit protocol. Library preparation and sequencing were performed on a NovaSeq 6000 platform (Illumina, Inc.) by Shanghai Biotechnology Corporation.

### 2.4 Single‐cell RNA‐seq data preprocessing and quality control

Cell Ranger (6.1.2, 10× Genomics) was used to produce a two‐dimensional count matrix containing the gene expressions for each cell, which was processed using the Seurat (4.1.0) package in R (4.1.3).^16^ Cells with gene counts lower than 500 or a mitochondrial gene ratio greater than 10% were omitted. However, in epithelial cells, the upper threshold for the mitochondrial gene ratio was set to 50% as these cells usually have higher mitochondrial content.^17^ Count matrices were normalized and integrated as in our previous study.^18^ Doublet cells were detected using the DoubletFinder (2.0.3) package, and only the cells labelled “Singlet” were retained.^19^

### 2.5 Single‐cell RNA‐seq data processing and integration

The NormalizeData function in the Seurat package was utilized for the normalization of the count matrix in each qualified sample. The ScaleData function was used for linear transformation and the RunPCA function was used to decrease the dimensions of the datasets (dimensions = 1:30). To minimize the potential batch effects generated in different runs, samples were integrated following the conventional pipeline.^18^ In brief, 2000 “anchors” genes were discovered among the samples using the FindIntegrationAnchors function with default parameters; then, these “anchors” genes were analysed using the IntegrateData function to eliminate batch effects and to output an integrated expression matrix.

### 2.6 Unsupervised clustering and cell annotation

As previously described,^18^, ^20^ the RunUMAP function in Seurat was used to reduce the dimensions of the dataset with a uniform manifold approximation and projection algorithm, while the FindNeighbors and FindClusters functions were used for clustering. We conducted two steps to comprehensively allocate the cell clusters.^20^ Major cell clusters were first identified through canonical markers, then each major cell type was subjected to a second round of clustering using higher resolutions.

### 2.7 Difference group enrichment analysis

We estimated the observed and expected number of cells in the clusters and quantitatively compared the concentration of immune cells among NCs, IRs, and INRs according to a previously described formula.^21^, ^22^ The formula is as follows: Ro/e = (observed/expected), where the expected number in each grid is generated using the chi‐square test.

### 2.8 Differentially‐expressed‐gene analysis

The FindMarkers function with all default parameters was applied to calculate differentially expressed gene (DEGs). The Wilcoxon rank sum test served as the method for comparing the data and the Bonferroni method was used for *P‐*value correction. Unless otherwise specified, in the comparison between two groups, genes with an absolute log2 fold change(|log2FC|)of more than .5 and adjusted *P*‐values of less than 0.05 were considered to have significant differences.

### 2.9 Cell communication analysis using CellPhoneDB

We used the “statistical_analysis” option in CellPhoneDB (v2.0.6) software^23^ to analyze the expression of ligand‐receptor pairs between follicular B/memory B‐cell clusters and T‐follicular helper (Tfh) cells. The number of ligand‐receptor co‐expression pairs meeting the threshold (*P* < 0.05 and mean > 1) was considered significant, and interactions of interest were illustrated.

### 2.10 Definition of functional scores

We collected a group of gene sets from the literature and calculated functional scores using the AddModuleScore function in the Seurat package to elucidate the functional characteristics of each cell type. Genes related to IgA survival, immunoglobulin production, endoplasmic reticulum‐associated degradation (ERAD), and unfolded protein response (UPR) in IgA^+^ plasma cells were defined as previously reported.^24^, ^25^ The genes in each set are listed in Table S1.

### 2.11 Transcriptional factor analysis

Transcriptional factor (TF) analysis was performed using the SCENIC package (v.1.2.4) and the Arboreto package (version 0.1.6).^26^ Briefly, the co‐expression modules in the normalized RNA‐seq expression matrix were examined. Meanwhile, regions up to 500 bp upstream of the transcription start site and ± 10 kbp around the transcription start site were examined for TF‐binding motifs with default parameters. Candidate binding elements were identified, and the transcriptional activity was calculated for each cell.

### 2.12 Trajectory analysis

The dynamics and regulators of B‐cell fate in the IRs and INRs were demonstrated by the pseudotemporal ordering of single cells using the Monocle 2 package in R.^27^, ^28^ Briefly, the top 1500 highly variable genes of B cells, excluding cycling B and germinal centre B cells, were subjected to a standard pipeline in Monocle 2 with default parameters, and the cells were ordered using the “order cells” function.

### 2.13 Multicolour immunohistochemistry staining

Fresh gut tissue was stabilized in a 4% formaldehyde solution and paraffin embedding. Multiplex immunofluorescence staining was performed as previously described.^20^ The paraffin‐embedded tissue was cut and placed into a 4‐μm‐thick glass slide. The slides were dewaxed in xylene for 30 min and then rehydrated twice with anhydrous ethanol for 5 min, 95% ethanol for 5 min, and 75% ethanol for 2 min. A microwave was used for heat‐induced epitope retrieval, during which the slides were immersed in a boiling ethylenediaminetetraacetic acid buffer (ZLI‐9079, Zsbio) for 15 min. An antibody diluent/block from AlphaX Bio was used for blocking. Immunohistochemistry (IHC) experiments were performed using Alpha Painter X30 and analyzed using the following primary antibodies: CD4 (ZA‐0519, OriGene), CD74 (ZM‐0290, OriGene), CD19 (ZM‐0038, OriGene), IgD (ZA‐0443, OriGene), IgA (ZA‐0446, OriGene), CD138(ZA‐0584, OriGene), and CXCR5 (ab254415, Abcam). The primary antibodies were all incubated for 1 h at 37°C. An AlphaTSA Multiplex IHC Kit (cat# AXT36100041) was used for visualization. After each staining cycle, heat‐induced epitope retrieval was conducted to remove all antibodies. The slides were counterstained for nuclei with 4′,6‐diamidino‐2‐phenylindole for 5 min and enclosed in a mounting medium. Scanned multispectral images were obtained using Axiosan 7 (ZEISS).

### 2.14 Sequencing analysis of faecal microbiota

To obtain faecal samples, disposable sterile bedpans, and tubes were prepared as previously described.^29^ The participants collected the interior portion of the feces and placed it into a tube. The freshly collected faecal samples were then frozen at −80°C.^29^ All of the faecal specimens were used together for 16S rRNA gene sequencing as follows. Microbial genomic DNA was extracted from the feces samples using the E.Z.N.A. soil DNA Kit (Omega Bio‐tek) following the instructions. The V3‐V4 of the bacterial 16S rRNA gene was amplified using an ABI GeneAmp 9700 PCR thermocycler (ABI) as previously reported.^29^ The raw sequencing reads of the 16S rRNA gene underwent demultiplexing and quality filtering using FASTP version 0.20.0, followed by merging with FLASH version 1.2.7. Subsequently, operational taxonomic units (OTUs) were clustered with a 97% similarity cutoff utilizing UPARSE version 7.1.^30^ Additionally, chimeric sequences were detected and subsequently removed. Furthermore, the taxonomic analysis of each representative OTU sequence was conducted using RDP Classifier version 2.2, referencing a 16S rRNA database (for instance, Silva version138), and employing a confidence threshold of .7.^31^

### 2.15 Flow cytometric analysis

Monoclonal antibodies were purchased from BD Biosciences. The peripheral blood mononuclear cells were dyed with APC‐Cyanine7‐anti‐CD45, BUV395‐anti‐CD3, Percp‐eFluor710‐anti‐CD4, PE‐Cy7‐anti‐CD8, APC‐anti‐CCR7, FITC‐anti‐CD45RA, BV421‐anti‐CD38, and PE‐anti‐HLA‐Dr antibodies. A BD FACSymphon A5 was used for the analysis.

### 2.16 Statistical analysis

Unless otherwise specified, non‐parametric tests were used for inter‐group comparisons. When the number of groups was two, the Wilcoxon test was used for comparisons through the “stat_compare_means” function in the “ggpubr” R package. When comparing multiple groups (*n* > 2), the “kwAllPairsDunnTest” function in the “PMCMRplus” package was used to conduct the Dunn method for pairwise comparisons between groups, and *P*‐values were corrected using the Benjamini–Hochberg method. The adjusted *P*‐value was considered significant at a threshold of .05. For the correlation analysis, Pearson's correlation coefficients were computed using the “cor” function in R. Results were fitted linearly, and 95% confidence intervals were derived.

## 3 RESULTS

### 3.1 Single‐cell transcriptomic analysis of gut mucosal cells in NCs, IRs, and INRs

**FIGURE 1:**
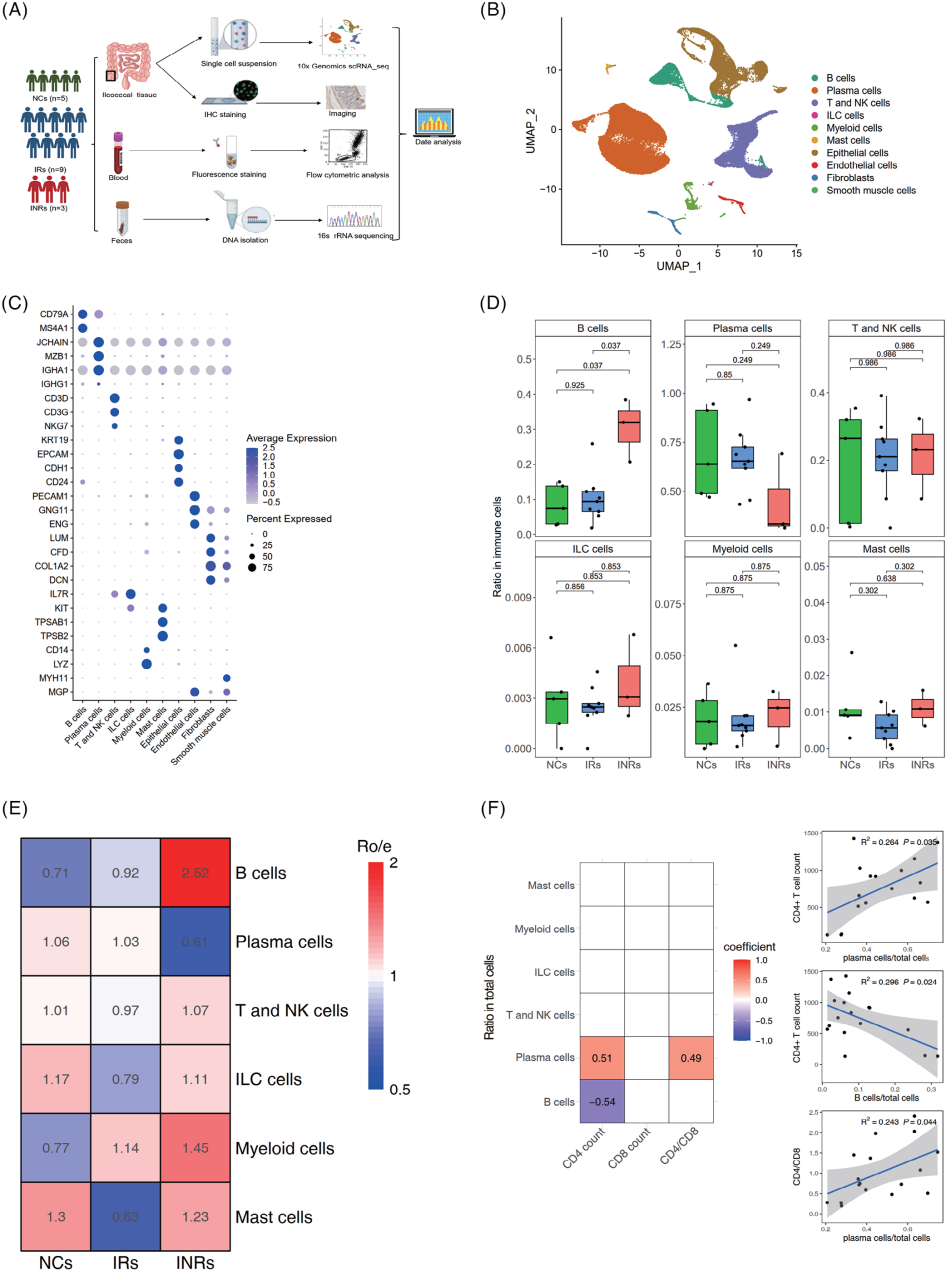
Study design and the single‐cell transcriptome map of intestinal tissues from the NCs, IRs, and INRs. (A) Experimental design and workflow. (B) UMAP plots displaying 10 major cell‐type clusters based on 68,345 cells of ileocecal mucosal tissues from five NCs, nine NRs, and three INRs. (C) Heatmap showing the expression levels of genes from the major cell‐type clusters identified in the gut mucosal tissue. (D) Boxplot showing the ratios of six major immune cell‐type clusters in the gut mucosal cells of each individual in the NC, IR, and INR groups. Horizontal lines represent median values, and conditions are shown in the farthest data point within a maximum of 1.5× interquartile range. (E) Enrichment scores of the major immune cell clusters in the NCs, IRs, and INRs were estimated based on the ratio of observed to expected cell numbers (Ro/e score). (F) Correlation analysis of the immune cell ratio with the peripheral blood CD4^+^ T‐cell count, CD8^+^ T‐cell count, and CD4/CD8 ratio.

We observed and characterized 54 475 immune cells and 13 870 non‐immune cells from the ileocecal mucosal tissues of 17 individuals, including NCs, IRs, and INRs (Figure 1A, Figure S1, and Table S2). Table 2 presents the clinical information of the enrolled individuals.

**TABLE 2:** Cell number of each enrolled person.

	B cells	Plasma cells	T‐ and NK cells	ILC cells	Myeloid cells	Mast cells	Epithelial cells	Endothelial cells	Fibroblasts	Smooth muscle cells
NC1	74	2458	36	4	98	24	948	76	143	22
NC2	741	6372	2637	66	50	106	14	57	8	11
NC3	559	1824	1195	11	105	34	565	15	33	20
NC4	63	1983	7	0	38	6	504	24	34	0
NC5	902	3072	2315	22	47	172	140	195	23	36
IR1	259	2469	756	13	75	17	647	33	58	10
IR2	268	2242	245	13	47	29	382	75	72	15
IR3	7	358	0	0	5	0	139	1	2	1
IR4	178	2167	870	8	69	21	696	86	38	10
IR5	242	1434	591	6	35	13	1532	83	45	17
IR6	373	1944	515	6	167	39	1957	74	630	56
IR7	428	1482	1275	7	37	30	709	59	50	11
IR8	49	542	139	2	12	2	678	2	47	4
IR9	1044	1752	1201	10	24	4	380	4	5	2
INR1	135	451	56	2	4	4	1515	1	0	0
INR2	983	965	994	6	76	49	237	62	91	18
INR3	848	737	512	15	72	24	383	45	28	12

According to gene expression, 10 major cell clusters were identified in this study (Figures 1B and C). They included B cells (*CD79A* and *MS4A1*), plasma cells (*JCHAIN, MZB1, IGHA1*, and *IGHG1*), T and NK cells (*CD3D, CD3G*, and *NKG7*), innate lymphoic cells (ILCs) (*IL7* and *KIT*), myeloid cells (*CD14* and *LYZ*), mast cells (*KIT*, *TPSAB*1, and *TPSB2*), epithelial cells (*KRT19*, *EPCAM*, *CDH1*, and *CD24*), endothelial cells (*PECAM1*, *GNG11*, and *ENG*), fibroblasts (*LUM*, *CFD*, *COL1A2*, and *DCN*) and smooth muscle cells (*MYH11* and *MGP*).

Subsequently, we analyzed the immune cell clusters that exhibited different patterns of cell enrichment between the IRs and INRs (Figure 1D and E; Figures S2A and B). B cells were abundantly present in the gut mucosal tissue of the INRs. Plasma cells exhibited preferential enrichment in the gut mucosal tissues of NCs and IRs compared with the INRs. ILC, myeloid cell, mast cells, and T‐ and NK‐cell numbers were consistent between the IRs and INRs. Further analysis revealed a positive association between the frequency of gut plasma cells and CD4^+^ T‐cell counts (*R* = .51, *P *= 0.035) and the ratio of CD4 to CD8 (*R* = .49, *P *= 0.044). The B‐cell count showed an inverse relationship with CD4^+^ T‐cell counts (*R* = −.54, *P *= 0.024) (Figure 1F). These results indicate that gut mucosal B cells significantly increased, while gut plasma cells significantly decreased in the INRs.

### 3.2 Enrichment of gut follicular B and memory B cells in INRs

**FIGURE 2:**
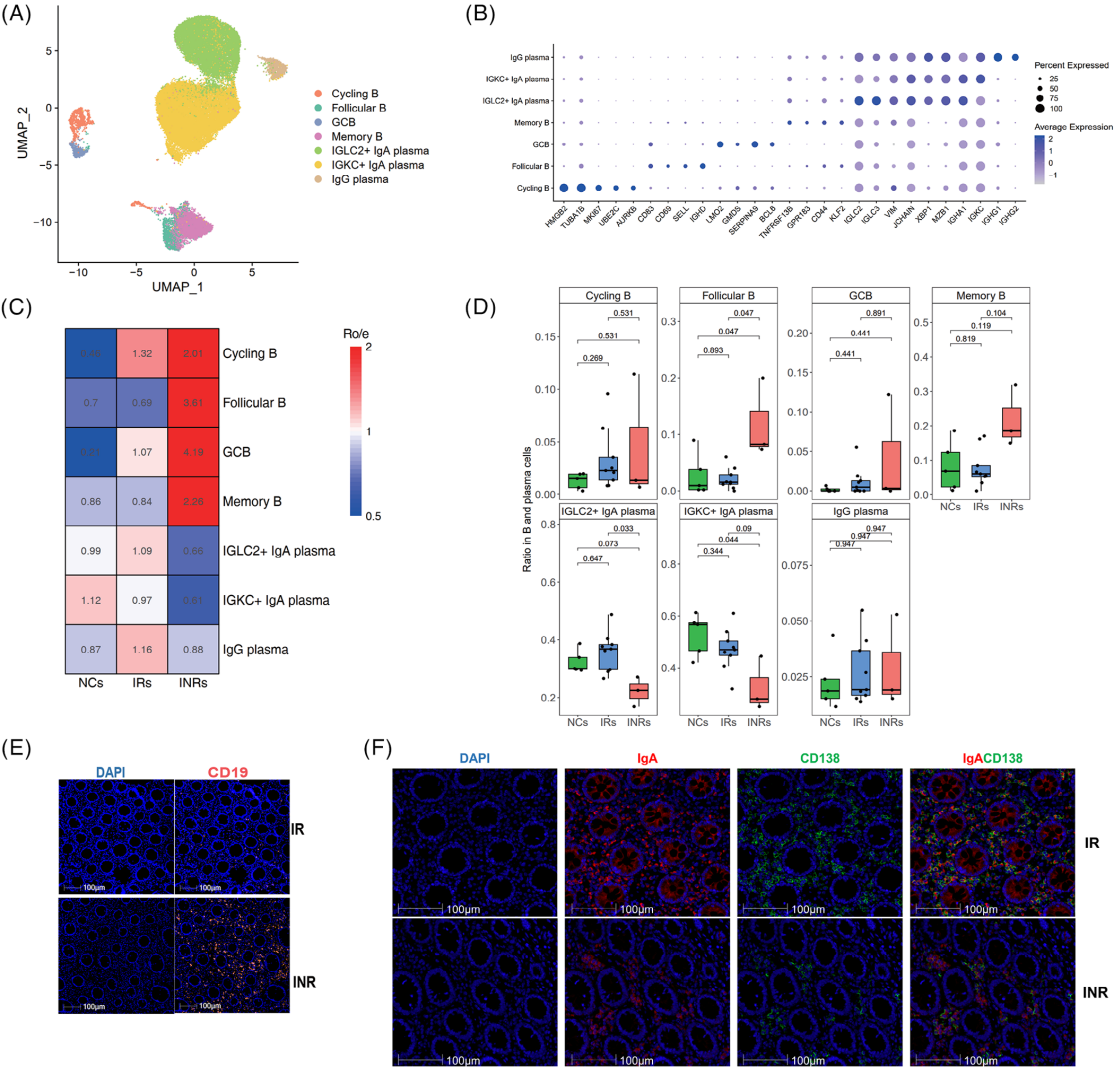
Subclustering of the B and plasma cell cluster identified in gut tissues from NCs, IRs, and INRs. (A) UMAP plot displaying seven distinct B‐cell subclusters, including three plasma cell subclusters. (B) Dot plot showing the average expression levels of marker genes and the percentage of cells expressing marker genes in each subcluster. (C) Enrichment scores of the seven B‐cell subclusters were estimated based on the ratio of observed to expected cell numbers (Ro/e score). (D) Boxplots representing the proportions of the seven B‐cell subclusters to the total B cells in the NCs, IRs, and INRs. (E) IHC staining of B cells (CD19^+^) in the IRs and INRs. Horizontal lines represent median values, and conditions are shown in the farthest data point within a maximum of 1.5× interquartile range. (F) IHC staining of plasma cells (CD138^+^) and IgA in the IRs and INRs. DAPI, 4′,6‐diamidino‐2‐phenylindole; IHC, immunohistochemistry.

Further analysis yielded seven subclusters of gut B and plasma cells, as shown in detail in Figure 2A and B. In general, the four gut B‐cell subsets exhibited preferential enrichment in the INRs compared with the IRs and NCs (Figure 2C and D and Figure S3A and B). In particular, follicular B and memory B cells exhibited significant enrichment in the gut mucosal tissue of the INRs compared with the IRs (Figure 2D). Simultaneously, the percentages of IGLC2^+^IgA^+^ and IGKC^+^IgA^+^ plasma cells were significantly decreased in the INRs (Figure 2D). No difference was detected in the percentage of IgG^+^ plasma cells between the IRs and INRs (Figure 2D). Furthermore, IHC staining showed an increase in B cells (CD19^+^), with a decrease in plasma (CD138^+^) and IgA cells in the INRs, as shown in Figure 2E and F. Therefore, gut follicular B and memory B cells were significantly increased while gut IgA^+^plasma cells were significantly reduced in the INRs.

### 3.3 Reduced potential in the differentiation of follicular or memory B cells into gut plasma cells in INRs

**FIGURE 3:**
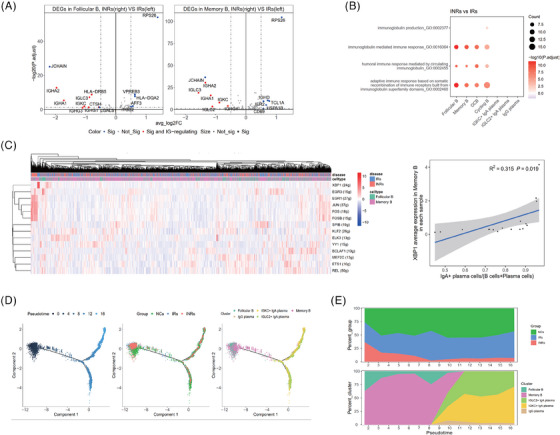
Abnormal cell trajectory of follicular/memory B‐cell differentiation into plasma cells in INRs. (A) DEGs of the INRs and IRs in follicular and memory B cells. (B) GO terms (BP) related to immunoglobulin regulation enriched in DEGs between INRs and IRs in the seven B subclusters. (C) Activity analysis of the transcription factors in follicular and memory B cells using the SCENIC package. The cells in the two distinct subclusters and two different clinical groups are annotated with different colors (left). Correlation between IgA^+^ plasma ratios to total B cells and the average expression of XBP1 in memory B cells (right). (D) Pseudotime trajectory showing the differentiation of follicular/memory B cells into plasma cells. Pseudotime (left), clinical groups (middle), and subclusters (right) were labeled with different colors. (E) Cell distribution in different groups (upper) and subclusters (lower) at each pseudotime point. BP, biological process; DEGs, differentially expressed genes; GO, gene ontology.

As a significant difference was found in the follicular and memory B cells of the IRs and INRs (Figure 2D), an analysis was necessary to determine whether a decrease in gut plasma cells was associated with a change in the differentiation of B cells in these patients. We identified significant changes in 28 DEGs of follicular and memory B cells between the IRs and INRs (Figure 3A; Table S2). Compared with those in the cells of IRs, most genes with low expression levels in the follicular and memory B cells of the INRs were mainly concentrated in immunoglobulin regulation‐related pathways, such as GO_0002460, which are involved in adaptive immune responses and are based on the somatic recombination of immune receptors (Figure 3A and B).

We further observed that the transcription factor *XBP1*, a key regulator of the differentiation of B cells into plasma cells, was highly activated in the follicular and memory B cells of the IRs and was positively associated with the ratio of IgA^+^ plasma cells (*R* = .561, *P *= 0.019) (Figure 3C). Transcription factors related to the complex AP‐1, including *JUN, FOS*, and *FOSB*, were strongly present in the follicular and memory B cells of the INRs (Figure 3C). Considering the essential role of XBP1 in the transformation of B cells into plasma cells^31^, ^32^ and the decreased expression level of *XBP1* in the INRs, we further used pseudotime trajectories to analyze the differences between the IRs and INRs during the differentiation of follicular and memory B cells into plasma cells (Figure 3D and E). In the trace, the starting branch was composed of follicular and memory B cells, whereas the plasma cells were represented by the other two branches (Figure 3D). As shown in Figure 3D and E, the lowest ratio of cells from the INRs was present at around the pseudotime point of 8−9 among the three groups (NCs, IRs, and INRs) (Figure 3E), which is the point at which B cells differentiate into plasma cells (Figure 3E). These findings suggest a reduced potential for the differentiation of follicular or memory B cells into plasma cells in INRs.

### 3.4 Reduced expression of CD74/MIF and CD74/COPA pairs in the memory‐B/Tfh cells of INRs

**FIGURE 4:**
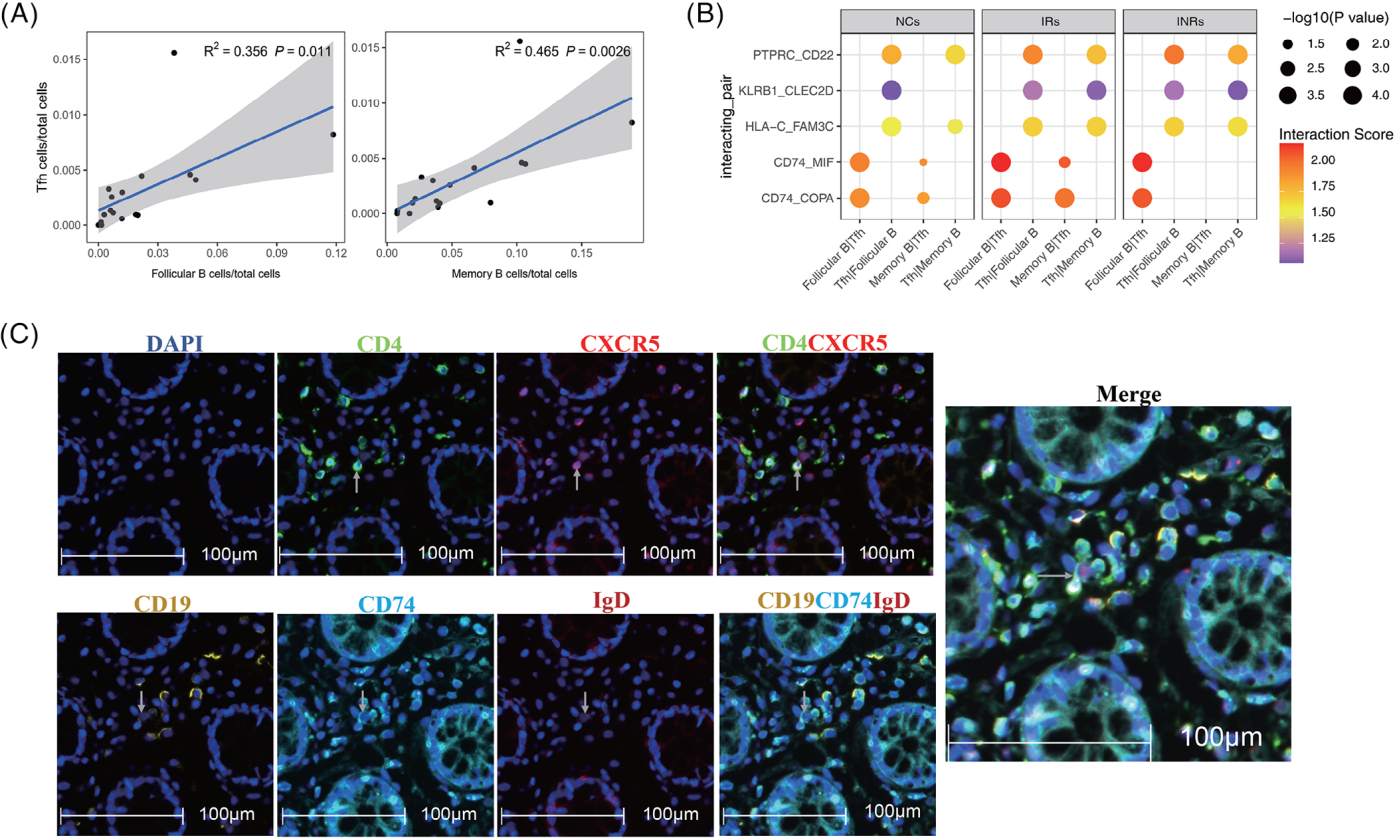
Interactions between Tfh and follicular/memory B cells. (A) Correlation of the Tfh cell ratio with the follicular (left) and memory (right) B‐cell ratios. All ratios were estimated based on the total immunocyte count. (B) Ligand‐receptor interactions in Tfh cells and follicular/memory B cells were predicted using CellPhoneDB with a threshold of mean > 1 and *P* < 0.05. (C) Multiplex IHC staining of CD74 of memory B (CD19^+^IgD^−^) cells in adjacent Tfh (CD4^+^CXCR5^+^) (from the IR patients).

Interactions between Tfh and B cells play a role in the differentiation of B cells into plasma cells.^33^ To investigate whether abnormal interactions occur between Tfh and follicular or memory B cells, we compared these interactions in the NCs, IRs, and INRs. First, we observed a positive correlation of Tfh with follicular (*R* = .597, *P *= 0.011) and memory B cells (R = .682, *P *= 0.0026) (Figure 4A). To analyze the potential interactions between Tfh and follicular or memory B cells, we used CellPhoneDB analysis to infer the cell–cell interactions of Tfh with follicular and memory B cells. The dot plot in Figure 4B shows the different strengths of potential ligand‐receptor pairs in follicular B/Tfh and memory B/Tfh interactions. The results of the analysis showed that CD74/MIF and CD74/COPA pairs had lower interaction potentials in the memory‐B/Tfh cells of the INRs compared with those of the NCs and IRs (Figure 4B). We further characterized the gut tissue sections by using multiplex IHC staining to confirm that Tfh cells (CD4^+^CXCR5^+^) were spatially adjacent to memory B cells (CD19^+^IgD^−^) expressing CD74 (Figure 4C). The expression level of the KLRB1‐CLEC2D pair in memory B/Tfh cells were significantly lower in the NCs than in IRs or INRs (Figure 4B).

### 3.5 Positive association of gut IGKC^+^IgA^+^ plasma cells with peripheral CD4^+^ T‐cell restoration

**FIGURE 5:**
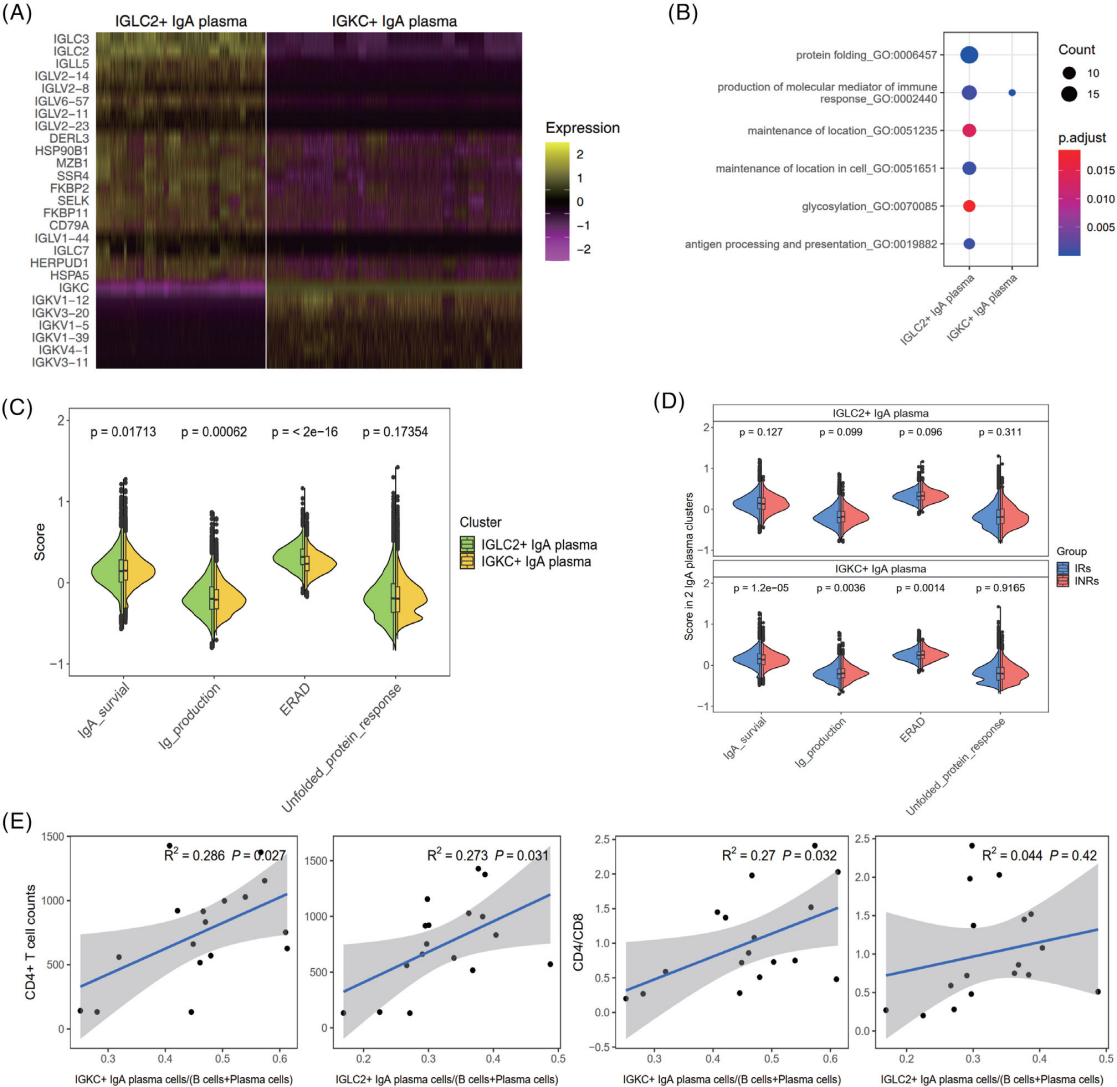
Differential characteristics of IGLC2^+^IgA^+^ plasma cells (IgA lambda subtype) and IGKC^+^IgA^+^ plasma cells (IgA kappa subtype). (A) Heatmap of the gene expression of the top 27 DEGs in the IGLC2^+^IgA^+^ and IGKC^+^IgA^+^ plasma cells. (B) The most significant level‐3 GO biological process terms for genes highly expressed in IGLC2^+^IgA^+^ and IGKC+IgA+ plasma cells. (C) Functional scores of genes associated with survival, immunoglobulin production, ERAD, and UPR in IGLC2^+^IgA^+^ and IGKC^+^IgA^+^ plasma cells. (D) Functional scores of IGLC2^+^IgA^+^ and IGKC^+^IgA^+^ plasma cells in the IRs and INRs. (E) Correlation of IgA^+^ plasma cell abundance in B cells with CD4^+^ T‐cell counts and CD4/CD8 ratios in the peripheral blood. DEGs, differentially expressed genes; ERAD, endoplasmic reticulum‐associated degradation; GO, gene ontology; UPR, unfolded protein response.

Considering the lack of extensive research on IGLC2^+^IgA^+^ and IGKC^+^IgA^+^ plasma cells, we performed an analysis to enhance our understanding of these two cellular subsets. To compare the functional differences between IGLC2^+^IgA^+^ and IGKC^+^IgA^+^ plasma cells, DEGs were analyzed. The analysis revealed a higher expression of genes in IGLC2^+^IgA^+^ plasma cells than in IGKC^+^IgA^+^ plasma cells (Figure 5A). Notably, genes highly expressed in IGLC2^+^IgA^+^ plasma cells were enriched in protein folding, the production of molecular mediators of the immune response, cellular localization maintenance, glycolysis, and antigen processing and presentation pathways (Figure 5B). In contrast, genes highly expressed in IGKC^+^IgA^+^ plasma cells were mostly involved in the production of molecular mediators (Figure 5B). Further analysis performed according to a previous study^24^ showed that IGKC^+^IgA^+^ plasma cells had higher survival scores than IGLC2^+^IgA^+^ plasma cells (Figure 5C), whereas IGLC2^+^IgA^+^ plasma cells had higher immunoglobulin production and ERAD scores (Figure 5C). When comparing the characteristics of the two cell groups between the IRs and INRs, we observed that IGKC^+^IgA^+^ plasma cells had higher survival scores in IRs than in INRs (Figure 5D). Additionally, IGKC^+^IgA^+^ plasma cells had higher levels of immunoglobulin and ERAD proteins in the INRs than in the IRs (Figure 5D). No significant differences were observed in the characteristics of IGLC2^+^IgA^+^ plasma cells between the IRs and the INRs (Figure 5D). Notably, the frequency of both IGKC^+^IgA^+^ (*R* = .535, *P *= 0.027) and IGLC2^+^IgA^+^ plasma cells (*R* = .522, *P *= 0.031) correlated with CD4^+^ T‐cell count, and only IGKC^+^IgA^+^ plasma cells were related to the CD4/CD8 ratio (*R* = .52, *P *= 0.032) (Figure 5E).

### 3.6 Negative association of IGKC^+^IgA^+^ plasma cells with *f. Prevotellaceae* abundance and T‐cell overactivation

**FIGURE 6:**
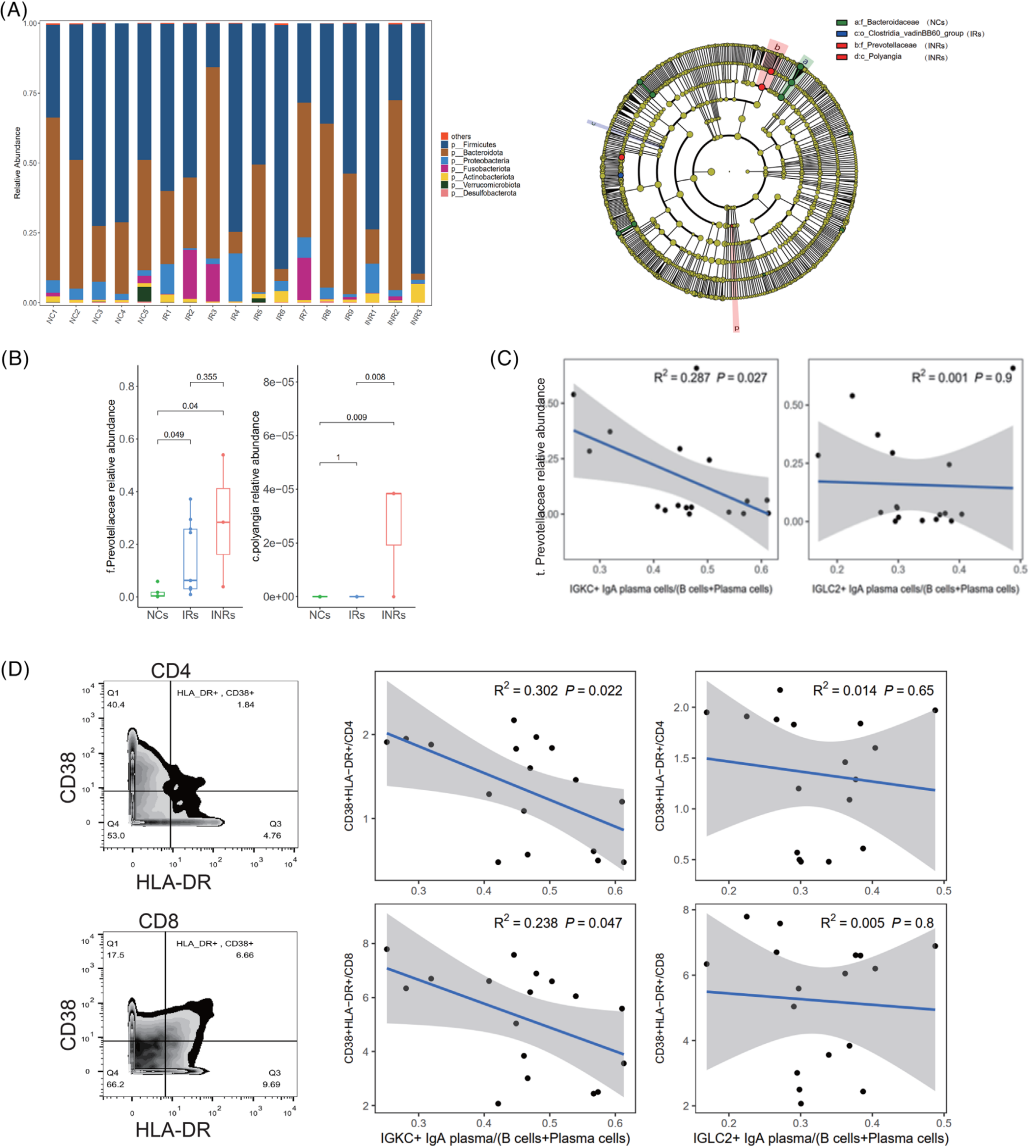
Correlation of IGLC2^+^IgA^+^ plasma cells (IgA lambda) and IGKC^+^IgA^+^ plasma cells (IgA kappa) with *f. Prevotellaceae* and immune activation. (A) The stacked bar represents the median relative abundance of the gut microbiota at the phylum level. Taxonomic cladograms derived from LEfSe of the 16S rRNA sequences. OTUs showing significant differences between groups are shaded in green (NCs), blue (IRs), or red (INRs). (B) Relative abundances of *f*. *Prevotellaceae* and *C. Polyangia* among the NCs, IRs, and INRs (mean with SD). (C) Relationship of *f*. *Prevotellaceae* and the proportions of IGKC^+^IgA^+^ and IGLC2^+^IgA^+^ plasma cells in total B cells and plasma cells. D Relationship between T‐cell activation (HLA‐DR^+^CD38^+^) and the proportions of IGKC^+^IgA^+^ and IGLC2^+^IgA^+^ plasma cells in total B cells and plasma cells.

To investigate the association between gut immune cells and microbiota, the faecal microbiota composition of the NCs, IRs, and INRs was analyzed. As shown in Figure 6A, the faecal microbiota in all three groups was mainly composed of the phyla *Firmicutes*, *Proteobacteria*, and *Bacteroidetes*. Notably, the IRs had a higher relative abundance of *Fusobacteria*, whereas the INRs had a higher relative abundance of *Actinobacteria*. Further analysis at lower taxonomic levels revealed that the family *Bacteroidaceae* was significantly enriched in the NCs, whereas the order *Clostridia_ Vadinbb60_Group* was enriched in the IRs. A notable increase was observed in the abundance of the family *Prevotellaceae* and class *Polyangia* in INRs compared with those in the IRs and NCs (Figure 6B). As IgA^+^ plasma cells may secrete IgA into the gastrointestinal mucosa and therefore play an essential role in symbiont homeostasis,^14^ we further analyzed the relationship of the relative abundance of *f. Prevotellaceae* with the frequencies of IGKC^+^IgA^+^ and IGLC2^+^IgA^+^ plasma cells. The frequency of IGKC^+^IgA^+^ plasma cells (*R* = −.536, *P *= 0.027), but not of IGLC2^+^IgA^+^ plasma cells (*R* = −.032, *P *= 0.9), was negatively associated with the relative abundance *f. Prevotellaceae* (Figure 6C). Similarly, we observed that the proportion of IGKC^+^IgA^+^ plasma cells (*R* = −.55, *P *= 0.022; *R* = −.488, *P *= 0.047 for the activation of CD4^+^ and CD8^+^ T cells, respectively), but not that of the IGLC2^+^IgA^+^ plasma cells (*R* = −.118, *P *= 0.65; *R* = −.071, *P *= 0.8 for the activation of CD4^+^ and CD8^+^ T cells, respectively), was negatively associated with the activation of both CD4^+^ and CD8^+^ T cells (HLA‐DR^+^CD38^+^) (Figure 6D).

### 3.7 Different profiles of gut and peripheral T‐cell subpopulations

Five CD4^+^ T‐cell clusters: CD4‐CCR7 (*CCR7*, *LEF1*, and *TCF7*), CD4‐ANXA1 (*ANXA1* and *CCL5*), Tregs (*ICOS*, *FOXP3*, and *TIGIT*), Th17 (*RORA* and *CCR6*) and Tfh(*PDCD1*, *CXCR5*, and *ID3*), were identified in the gut mucosal tissues (Figures S4A, B, and C). A comparison of the gut CD4^+^ T‐cell subpopulations among the IRs, INRs, and NCs showed no noteworthy differences in the frequencies of the other subsets among the NCs, IRs, or INRs (Figure S4D, E, and F). Peripheral T‐cell subsets were simultaneously analyzed, and no difference in the CD4/CD8 ratio was observed in the gut mucosal tissues; nevertheless, the peripheral CD4/CD8 ratio was significantly reduced in the INRs compared to that in the IRs (Figure S4G). In addition, a significant reduction in the peripheral naïve CD4^+^ T‐cell subsets of the INRs was observed (Figure S4H). However, peripheral effector memory (EM) subsets showed an increasing trend (Figure S4H). No significant differences were observed in the peripheral CM and EMRA subsets of CD4^+^ T cells among the three groups (Figure S4H).

Furthermore, seven subclusters of CD8^+^ T cells were identified: CD8‐ENTPD‐1 (*FCER1G*, *KLRC1*, *IKZF2*, *ID3*, *KIR2DL4*, *KLRC2*, and *TRDC*), CD8‐CCR6 (*NCR3*, *SLAMF1*, *GADD45G*, *TNFSF13B*, and *TNF*), CD8‐ZNF683 (*LINC00152*, *ALOX5AP*, and *COTL1*), CD8‐GZMK (*KLRG1* and *GZMK*), CD8‐CX3CR1 (*GNLY*, *PLEK*, *GZMH*, *FCGR3A*, *CX3CR1*, and *FGFBP1*), CD8‐IL7R (*FOS*, *RORA*, *MYBL1*, and *IL7R*), and CD8‐LET1 (*LDHB*, *NOSID*, *AIF1*, *TCF7*, *LEF1*, *SELL*, and *CCR7*) (Figure S5A, B, and C). Although some differences were observed in the gut CD8+ T‐cell subgroups between the IRs and INRs (Figure S5D, E, and F), no statistically significant difference was found among the subgroups, which was likely associated with the small sample size (Figure S5F). Conversely, the peripheral naïve CD8^+^ T‐cell subgroup significantly decreased in INRs, whereas the EM subgroup significantly increased (Figure S5G). No noteworthy difference was found in the CM CD8^+^ T‐cell subgroup among the three groups (Figure S5G). The counts of the EMRA CD8^+^ T‐cell subgroup displayed a notable elevation in the IRs when compared to the NCs (Figure S5G). Three gut mucosal myeloid subclusters: DC_CLEC9A (*S100B*, *CADM1*, *CLEC9A*, and *HLA‐DPB1*), DC_CD1C (*FCER1A*, *CD1C*, *CLEC10A*, and *HLA‐DPB1*), and macrophages (*SEPP1*, *CD68*, and *CD163*) were observed (Figure S6A, B, and C). Although the myeloid cell subgroups had some variation (Figure S6D, E, and F), no statistically significant difference was observed between the different subgroups among the IRs, INRs, and NCs (Figure S6F).

## 4 DISCUSSION

The gut immune system, which consists of gut mucosal tissues with more than 80% human lymphocytes and the intestinal‐associated lymphoid tissue with approximately 60% CD4^+^ T lymphocytes, is a major target of an HIV attack.^34^ The depletion of gut CD4^+^ T cells can persist throughout the course of the disease.^7^ However, the consequences of such a depletion of gut immune function, as well as the potential influence of ART on immune reconstitution, remain incompletely understood. Here, for the first time, we present the landscape and characteristics of gut mucosal immune cells in PLWH receiving ART at a single‐cell level. Our study identified six major gut immune cell clusters and demonstrated that the ratio of B cells, particularly follicular and memory B cells, was significantly elevated, whereas the ratio of plasma cells, specifically IGLC2^+^IgA^+^ and IGKC^+^IgA^+^ plasma cells, was obviously lower in the INRs.

Our study identified an abnormal distribution of B and plasma cell compartments in the gut mucosal tissues of the INRs. Correspondingly, the INRs exhibited an aberrant cell transition trajectory from follicular/memory B cells to plasma cells. This aberration may reflect a reduced potential for the differentiation of follicular/memory B cells into plasma cells and an essential characteristic of poor immune restoration in the INRs. To investigate the mechanism underlying the putative obstacle to the differentiation of follicular/memory B cells into plasma cells, we found that several crucial transcription factors, such as XBP1, which may determine follicular/memory B‐cell fate, were significantly differentially expressed between the INRs and IRs. This transcription factor is a fundamental regulator for the development of B cells into plasma cells.^25^ Thus, the reduced expression level of XBP1 in INRs may partially account for the reduced potential for the differentiation of follicular/memory B cells into plasma cells. In addition, genes related to immunoglobulin regulatory pathways had decreased expression levels in the INRs compared with the IRs. Notably, the GO_0002460 pathway represents an adaptive immune response. Therefore, the decreased expression level of the GO_0002460 pathway may also be linked to the putatively reduced ability to differentiate follicular/memory B cells into plasma cells via BCR signalling. Furthermore, as the somatic recombination of B cells and the subsequent Darwinian affinity‐driven selection of immunoglobulins require interactions between Tfh cells and B cells at germinal centres, the reduced strength of the interactions between CD74 from memory B cells and MIF/COPA from memory B/Tfh cells may be associated with the potential downregulation of B‐cell differentiation. CD74 is predominantly expressed on antigen‐presenting cells and serves as a crucial intracellular chaperone for MHC Class II. The interaction between CD74 and MIF, its natural ligand, is vital for initiating a sequence of signals that ultimately leads to an increase in BCL‐2 levels and subsequent influence on the differentiation and activation of memory B cells.^35^ Furthermore, an increase in the interactions between KLRB1 (CD161) from Tfh cells and CLEC2D (LLT1) from memory B cells was inferred in both the IRs and INRs compared to that the NCs. However, LLT1 remains expressed in early plasmablasts and is absent in memory B cells and plasma cells.^36^ Thus, a strong KLRB1‐CLEC2D interaction may also influence the phenotype and differentiation capability of memory B cells.

Plasma cells synthesize and secrete large amounts of specific antibodies, all of which must be correctly folded and posttranslationally modified within the endoplasmic reticulum (ER). ERAD serves as a fundamental quality control mechanism that identifies and transports terminally misfolded or unfolded proteins within the endoplasmic reticulum, which can cause upregulated cell apoptosis.^25^, ^37^ As ERAD activity was upregulated in IGKC^+^IgA^+^ plasma cells in the INRs compared with the IRs, ER stress‐induced apoptosis may result in a decrease in the abundance of IGKC^+^IgA^+^ plasma cells in INRs.

IgA^+^ plasma cells are predominantly found in the lamina propria of the GIT.^38^ Gut IgA^+^ plasma cells secrete microbe‐specific high‐affinity IgA daily against invading bacteria that populate the GIT. The results of the current study revealed that gut B cells are inversely related to CD4^+^ T‐cell counts, whereas plasma cells, especially the IGKC^+^IgA^+^ plasma cell subpopulation, were found to have a positive association with CD4^+^ T‐cell counts and the CD4/CD8 ratio but a negative one with the overactivation of T cells. Therefore, the altered distribution of B/plasma cells observed in the INRs may have essential clinical implications for future studies.^34^ In addition, four of the nine IRs were treated with integrase inhibitors. Among the three INRs, one was treated with an integrase inhibitor. The application rate of integrase inhibitors is significantly higher in IRs than in INRs. It is worth exploring whether integrase inhibitors are beneficial for immune recovery in the intestinal mucosa.

Damage to the intestinal epithelial barrier and subsequent microbial translocation are crucial factors that affect the degree of immune restoration after ART.^39^ A meta‐analysis showed that HIV infection leads to a reduction in intestinal microbial diversity.^40^ This study revealed an increase in faecal *Prevotellaceae* abundance in INRs, which was associated with a reduction in gut IGKC^+^IgA^+^ plasma cells. This is consistent with the partial restoration of intestinal microbial balance in late‐treated PLWH.^41^ *Prevotella* abundance has been positively related to the overactivation of mucosal T cells and myeloid DCs.^4^ *Prevotella* is also associated with inflammation, opportunistic infections, and autoimmune diseases.^42^ In another study, the abundance of *Bacteroides* was significantly reduced in both IRs and INRs, and this reduction may potentially be linked to systemic immune activation and chronic inflammation in vivo.^34^ Collectively, the decrease in gut IGKC^+^IgA^+^ plasma cell levels in INRs may further diminish IgA production and delay the restoration of the gut mucosal barrier, thereby promoting host immune overactivation and microbial translocation. The gut microbiome plays a key role in the gut–brain axis,^43^, ^44^ and whether *Prevotellaceae is* associated with neurodamage in INRs must be explored. In addition, in untreated chronic PLWH, ILCs may lead to intestinal mucosal inflammation and epithelial barrier disruption, which differs from the conditions in the current study in that there is a decrease in IgA^+^ plasma levels, and this difference may potentially be attributed to the disruption of the intestinal microenvironment^45^ and the difference in the patients enrolled.

This study had several limitations. First, this study included only three cases of INRs and no cases of untreated patients as controls. Second, we took tissue samples from the ileocecal region only, although some studies have shown in the SIV/SHIV rhesus monkey model that B cells and plasma cells in different parts of the intestine are similar.^46^ Further confirmation is required to prove whether our study results indicate the same changes in other parts of the intestine. Previously, faecal microbiota has been linked to gender and sexual orientation in addition to HIV infection.^47^ However, all of our study participants were men, representing a significant proportion of the PLWH population in China. Gay men, due to their tendency to engage in unprotected receptive anal intercourse, may exhibit a unique composition of human faecal microbiota, as previously reported.^42^ Consistent with prior research,^42^ our analysis revealed altered abundances of *Bacteroides*, *Prevotella*, and *Clostridium* in the feces of those MSM patients on ART. Since this study lacked an MSM control group without HIV infection, we were unable to ascertain whether the increase in faecal *Prevotellaceae* abundance observed in the INRs was due to MSM behaviour or a direct effect of HIV infection. Thus, the findings in this study may not apply to heterosexual male patients. In addition, female sex hormones such as estradiol,^48^ estrogen, and progesterone,^49^ also contribute to B‐cell function by activating the Tfh/B‐cell axis. Thus, the results of this study may not be generalizable to female cases. Despite these challenges, we posit that the IgA^+^ plasma cell characteristic in the male PLWH described here may have clinical implications for HIV therapy. Nevertheless, larger‐scale studies that include female participants are necessary to validate our findings and generalize them to a broader population of PLWH.

To conclude, this is the first study to describe the landscape of the gut mucosal immune cells in PLWH receiving ART at single‐cell resolution. Contrary to changes observed in the peripheral blood of INRs reported in a previous study,^8^ as shown in Figure 7 (Graphical Abstract), INRs exhibit a significant reduction in gut IGKC^+^IgA^+^ plasma cells, and the reduction in gut IGKC^+^IgA^+^ plasma cells is closely associated with a reduced potential for differentiation of follicular/memory B cells into plasma cells. Our findings provide insight into the pathological characteristics of the gut immune response and gut immune restoration in PLWH undergoing ART. In particular, given that underlying mechanisms remain poorly understood, conducting further in‐depth studies with a large sample size of PLWH is necessary to elucidate the role of gut mucosal immunity in the disease pathogenesis.

## AUTHOR CONTRIBUTIONS

All authors have contributed substantially to this work. We have approved the manuscript, and have agreed to this submission.

## CONFLICT OF INTEREST STATEMENT

The authors declare no conflict of interest.

## ETHICAL APPROVAL

The study protocol was approved by the Ethics Committee of the Fifth Medical Center of the Chinese PLA General Hospital (KY‐2021‐7‐6‐1).

## Supporting information

Supporting Information

Supporting Information

Supporting Information

Supporting Information

Supporting Information

Supporting Information

## Data Availability

The raw data for single‐cell RNA sequencing reported in this publication can be accessed under the Chinese Academy of Sciences (GSA‐Human: HRA007250) and are publicly accessible at https://ngdc.cncb.ac.cn/gsa‐human.

## References

[1] Lorvik KB , Meyer‐Myklestad MH , Kushekar K , et al. Enhanced gut‐homing dynamics and pronounced exhaustion of mucosal and blood CD4(+) T cells in HIV‐infected immunological non‐responders. Front Immunol. 2021;12:744155. 34691047 10.3389/fimmu.2021.744155 PMC8529151

[2] Brenchley JM , Price DA , Douek DC . HIV disease: fallout from a mucosal catastrophe? Nat Immunol. 2006;7(3):235‐239. 16482171 10.1038/ni1316

[3] Liu J , Wang L , Hou Y , et al. Immune restoration in HIV‐1‐infected patients after 12 years of antiretroviral therapy: a real‐world observational study. Emerg Microbes Infect. 2020;9(1):2550‐2561. 33131455 10.1080/22221751.2020.1840928 PMC7733958

[4] Yang X , Su B , Zhang X , Liu Y , Wu H , Zhang T . Incomplete immune reconstitution in HIV/AIDS patients on antiretroviral therapy: challenges of immunological non‐responders. J Leukoc Biol. 2020;107(4):597‐612. 31965635 10.1002/JLB.4MR1019-189R PMC7187275

[5] AIDS and Hepatitis C Professional Group , Society of Infectious Diseases , Chinese Medical Association ; Chinese Center for Disease Control and Prevention . Chinese Guidelines for the Diagnosis and Treatment of HIV/AIDS (2021 Edition). Infect Dis Immun. 2022;2(3):145‐167.

[6] Allers K , Puyskens A , Epple H‐J , et al. The effect of timing of antiretroviral therapy on CD4+ T‐cell reconstitution in the intestine of HIV‐infected patients. Mucosal Immunol. 2016;9(1):265‐274. 26129649 10.1038/mi.2015.58

[7] Douek DC , Roederer M , Koup RA . Emerging concepts in the immunopathogenesis of AIDS. Annu Rev Med. 2009;60:471‐484. 18947296 10.1146/annurev.med.60.041807.123549 PMC2716400

[8] Li H , Tang Y , Wang Y , et al. Single‐cell sequencing resolves the landscape of immune cells and regulatory mechanisms in HIV‐infected immune non‐responders. Cell Death Dis. 2022;13(10):849. 36195585 10.1038/s41419-022-05225-6 PMC9532384

[9] Guo X‐Y , Guo Y‐T , Wang Z‐R , et al. Severe intestinal barrier damage in HIV‐infected immunological non‐responders. Heliyon. 2023;9(10):e20790. 37876458 10.1016/j.heliyon.2023.e20790 PMC10590933

[10] Hong JJ , Amancha PK , Rogers K , Ansari AA , Villinger F . Spatial alterations between CD4(+) T follicular helper, B, and CD8(+) T cells during simian immunodeficiency virus infection: t/B cell homeostasis, activation, and potential mechanism for viral escape. J Immunol. 2012;188(7):3247‐3256. 22387550 10.4049/jimmunol.1103138 PMC3311732

[11] Guo Y‐T , Guo X‐Y , Fan L‐N , et al. The imbalance between intestinal Th17 and Treg cells is associated with an incomplete immune reconstitution during long‐term antiretroviral therapy in patients with HIV. Viral Immunol. 2023;36(5):331‐342. 37184871 10.1089/vim.2023.0017

[12] Moretti S , Schietroma I , Sberna G , et al. HIV‐1‐host interaction in gut‐associated lymphoid tissue (GALT): effects on local environment and comorbidities. Int J Mol Sci. 2023;24(15):12193. 37569570 10.3390/ijms241512193 PMC10418605

[13] Chen K , Magri G , Grasset EK , Cerutti A . Rethinking mucosal antibody responses: IgM, IgG and IgD join IgA. Nat Rev Immunol. 2020;20(7):427‐441. 32015473 10.1038/s41577-019-0261-1 PMC10262260

[14] Fahlgren A , Hammarström S , Danielsson Å , Hammarström M‐L . Increased expression of antimicrobial peptides and lysozyme in colonic epithelial cells of patients with ulcerative colitis. Clin Exp Immunol. 2003;131(1):90‐101. 12519391 10.1046/j.1365-2249.2003.02035.x PMC1808590

[15] Huang EH , Hynes MJ , Zhang T , et al. Aldehyde dehydrogenase 1 is a marker for normal and malignant human colonic stem cells (SC) and tracks SC overpopulation during colon tumorigenesis. Cancer Res. 2009;69(8):3382‐3389. 19336570 10.1158/0008-5472.CAN-08-4418 PMC2789401

[16] Hao Y , Hao S , Andersen‐Nissen E , et al. Integrated analysis of multimodal single‐cell data. Cell. 2021;184(13):3573‐3587. e29. 34062119 10.1016/j.cell.2021.04.048 PMC8238499

[17] Zhang L , Li Z , Skrzypczynska KM , et al. Single‐cell analyses inform mechanisms of myeloid‐targeted therapies in colon cancer. Cell. 2020;181(2):442‐459. e29. 32302573 10.1016/j.cell.2020.03.048

[18] Zhang J‐Y , Wang X‐M , Xing X , et al. Single‐cell landscape of immunological responses in patients with COVID‐19. Nat Immunol. 2020;21(9):1107‐1118. 32788748 10.1038/s41590-020-0762-x

[19] Mcginnis CS , Murrow LM , Gartner ZJ . DoubletFinder: doublet detection in single‐cell RNA sequencing data using artificial nearest neighbors. Cell Syst. 2019;8(4):329‐337. e4. 30954475 10.1016/j.cels.2019.03.003 PMC6853612

[20] Zhang C , Li J , Cheng Y , et al. Single‐cell RNA sequencing reveals intrahepatic and peripheral immune characteristics related to disease phases in HBV‐infected patients. Gut. 2023;72(1):153‐167. 35361683 10.1136/gutjnl-2021-325915 PMC9763233

[21] Guo X , Zhang Y , Zheng L , et al. Global characterization of T cells in non‐small‐cell lung cancer by single‐cell sequencing. Nat Med. 2018;24(7):978‐985. 29942094 10.1038/s41591-018-0045-3

[22] Zhang L , Yu X , Zheng L , et al. Lineage tracking reveals dynamic relationships of T cells in colorectal cancer. Nature. 2018;564(7735):268‐272. 30479382 10.1038/s41586-018-0694-x

[23] Efremova M , Vento‐Tormo M , Teichmann SA , Vento‐Tormo R . CellPhoneDB: inferring cell‐cell communication from combined expression of multi‐subunit ligand‐receptor complexes. Nat Protoc. 2020;15(4):1484‐1506. 32103204 10.1038/s41596-020-0292-x

[24] Liu X , Yao J , Zhao Y , Wang J , Qi H . Heterogeneous plasma cells and long‐lived subsets in response to immunization, autoantigen and microbiota. Nat Immunol. 2022;23(11):1564‐1576. 36316480 10.1038/s41590-022-01345-5

[25] Trezise S , Nutt SL . The gene regulatory network controlling plasma cell function. Immunol Rev. 2021;303(1):23‐34. 34109653 10.1111/imr.12988

[26] Aibar S , González‐Blas CB , Moerman T , et al. SCENIC: single‐cell regulatory network inference and clustering. Nat Methods. 2017;14(11):1083‐1086. 28991892 10.1038/nmeth.4463 PMC5937676

[27] Trapnell C , Cacchiarelli D , Grimsby J , et al. The dynamics and regulators of cell fate decisions are revealed by pseudotemporal ordering of single cells. Nat Biotechnol. 2014;32(4):381‐386. 24658644 10.1038/nbt.2859 PMC4122333

[28] Qiu X , Hill A , Packer J , Lin D , Ma Y‐A , Trapnell C . Single‐cell mRNA quantification and differential analysis with Census. Nat Methods. 2017;14(3):309‐315. 28114287 10.1038/nmeth.4150 PMC5330805

[29] Guo X , Wang Z , Qu M , et al. Abnormal blood microbiota profiles are associated with inflammation and immune restoration in HIV/AIDS individuals. mSystems. 2023;8(5):e0046723. 37698407 10.1128/msystems.00467-23 PMC10654078

[30] Edgar RC . UPARSE: highly accurate OTU sequences from microbial amplicon reads. Nat Methods. 2013;10(10):996‐998. 23955772 10.1038/nmeth.2604

[31] Wang Q , Garrity GM , Tiedje JM , Cole JR . Naive Bayesian classifier for rapid assignment of rRNA sequences into the new bacterial taxonomy. Appl Environ Microbiol. 2007;73(16):5261‐5267. 17586664 10.1128/AEM.00062-07 PMC1950982

[32] Zhao J , Li L , Feng X , et al. TIGIT‐Fc fusion protein alleviates murine lupus nephritis through the regulation of SPI‐B‐PAX5‐XBP1 axis‐mediated B‐cell differentiation. J Autoimmun. 2023;139:103087. 37481835 10.1016/j.jaut.2023.103087

[33] Feng H , Zhao Z , Dong C . Adapting to the world: the determination and plasticity of T follicular helper cells. J Allergy Clin Immunol. 2022;150(5):981‐989. 36336399 10.1016/j.jaci.2022.09.018

[34] Geng S‐T , Zhang Z‐Y , Wang Y‐X , et al. Regulation of gut microbiota on immune reconstitution in patients with acquired immunodeficiency syndrome. Front Microbiol. 2020;11:594820. 33193273 10.3389/fmicb.2020.594820 PMC7652894

[35] Ramonell RP , Brown M , Woodruff MC , et al. Single‐cell analysis of human nasal mucosal IgE antibody secreting cells reveals a newly minted phenotype. Mucosal Immunol. 2023;16(3):287‐301. 36931600 10.1016/j.mucimm.2023.02.008 PMC11227847

[36] Llibre A , López‐Macías C , Marafioti T , et al. LLT1 and CD161 expression in human germinal centers promotes B cell activation and CXCR4 downregulation. J Immunol. 2016;196(5):2085‐2094. 26829983 10.4049/jimmunol.1502462 PMC4760235

[37] Chandran A , Oliver HJ , Rochet JC . Role of NFE2L1 in the regulation of proteostasis: implications for aging and neurodegenerative diseases. Biology (Basel). 2023;12(9):1169. 37759569 10.3390/biology12091169 PMC10525699

[38] Onabajo OO , Mattapallil JJ . Gut microbiome homeostasis and the CD4 T‐follicular helper cell IgA axis in human immunodeficiency virus infection. Front Immunol. 2021;12:657679. 33815419 10.3389/fimmu.2021.657679 PMC8017181

[39] Wang X‐M , Zhang J‐Y , Xing X , et al. Global transcriptomic characterization of T cells in individuals with chronic HIV‐1 infection. Cell Discov. 2022;8(1):29. 35351857 10.1038/s41421-021-00367-x PMC8964811

[40] Tuddenham SA , Koay WLA , Zhao N , et al. The impact of human immunodeficiency virus infection on gut microbiota alpha‐diversity: an individual‐level meta‐analysis. Clin Infect Dis. 2020;70(4):615‐627. 30921452 10.1093/cid/ciz258 PMC7319268

[41] Olivas‐Martínez I , Rosado‐Sánchez I , Cordero‐Varela JA , et al. Partial restoration of gut‐mucosal dysbiosis in late‐treated HIV‐infected subjects with CD4 T‐cell recovery. Clin Transl Med. 2022;12(4):e788. 35384348 10.1002/ctm2.788 PMC8982320

[42] Li K , Deng J , Zhang C , Lai G , Xie B , Zhong X . Gut microbiome dysbiosis in men who have sex with men increases HIV infection risk through immunity homeostasis alteration. Front Cell Infect Microbiol. 2023;13:1260068. 38035339 10.3389/fcimb.2023.1260068 PMC10687210

[43] Panther EJ , Dodd W , Clark A , Lucke‐Wold B . Gastrointestinal microbiome and neurologic injury. Biomedicines. 2022;10(2):500. 35203709 10.3390/biomedicines10020500 PMC8962360

[44] Nwafor DC , Brichacek AL , Foster CH , et al. Pediatric traumatic brain injury: an update on preclinical models, clinical biomarkers, and the implications of cerebrovascular dysfunction. J Cent Nerv Syst Dis. 2022;14:11795735221098125. 35620529 10.1177/11795735221098125 PMC9127876

[45] Dillon SM , Castleman MJ , Frank DN , et al. Brief report: inflammatory colonic innate lymphoid cells are increased during untreated HIV‐1 infection and associated with markers of gut dysbiosis and mucosal immune activation. J Acquir Immune Defic Syndr. 2017;76(4):431‐437. 28825942 10.1097/QAI.0000000000001523 PMC5659896

[46] Demberg T , Mohanram V , Venzon D , Robert‐Guroff M . Phenotypes and distribution of mucosal memory B‐cell populations in the SIV/SHIV rhesus macaque model. Clin Immunol. 2014;153(2):264‐276. 24814239 10.1016/j.clim.2014.04.017 PMC4086409

[47] Noguera‐Julian M , Rocafort M , Guillén Y , et al. Gut microbiota linked to sexual preference and HIV infection. EBioMedicine. 2016;5:135‐146. 27077120 10.1016/j.ebiom.2016.01.032 PMC4816837

[48] Valeff NJ , Ventimiglia MS , Diao L , Jensen F . Lupus and recurrent pregnancy loss: the role of female sex hormones and B cells. Front Endocrinol (Lausanne). 2023;14:1233883. 37859991 10.3389/fendo.2023.1233883 PMC10584304

[49] Monteiro C , Kasahara T , Sacramento PM , et al. Human pregnancy levels of estrogen and progesterone contribute to humoral immunity by activating T(FH)/B cell axis. Eur J Immunol. 2021;51(1):167‐179. 33012073 10.1002/eji.202048658

