# Supplementary figures and images for: Landscape of gut mucosal immune cells showed gap of follicular or memory B cells into plasma cells in immunological non‐responders
Source: Clin Transl Med. 2024 May 23;14(5):e1699. doi: 10.1002/ctm2.1699 (PMC11116468; doi:10.1002/ctm2.1699)

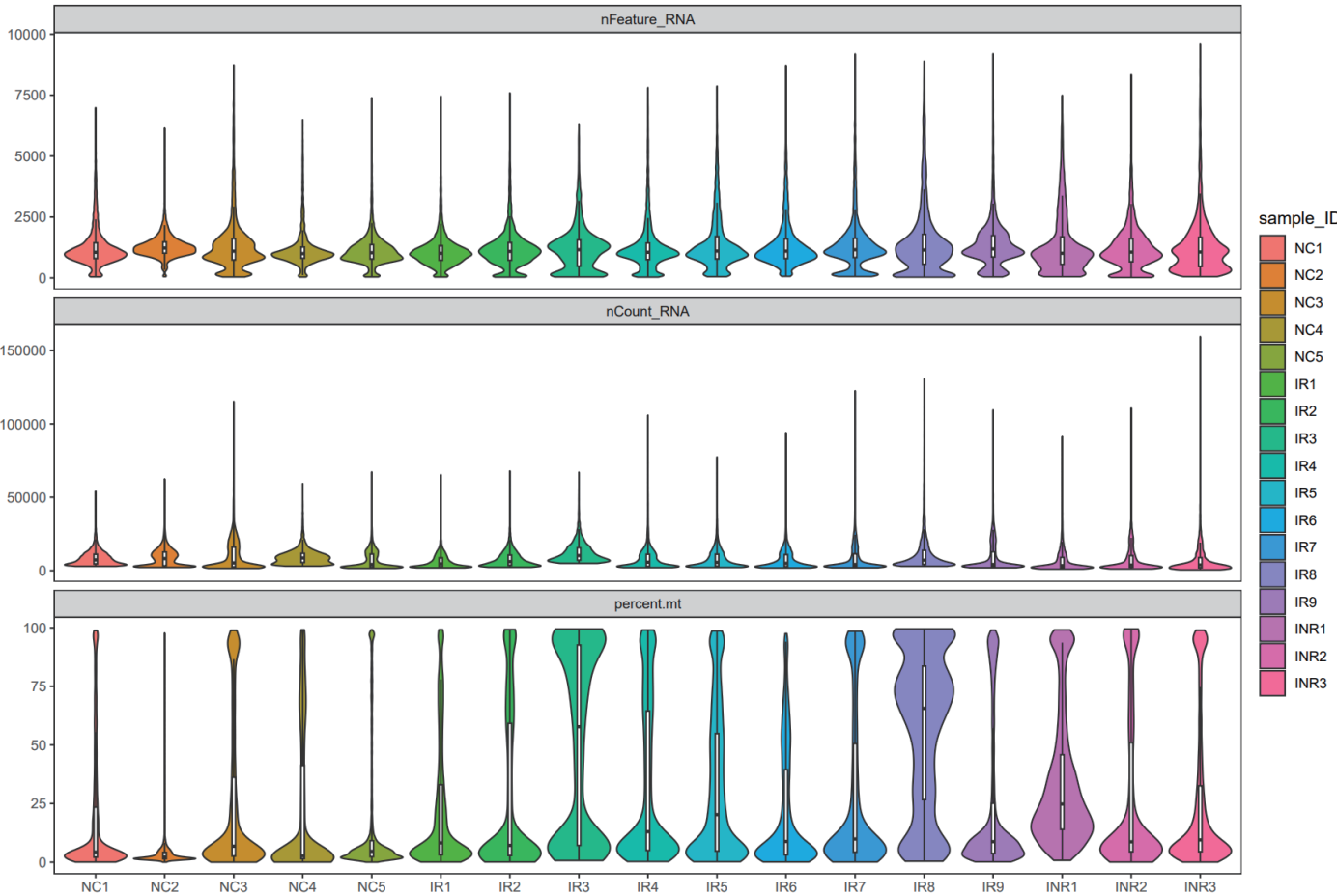

Supplement: Supplementary file 1 — Supporting Information [file CTM2-14-e1699-s006.pdf]

A

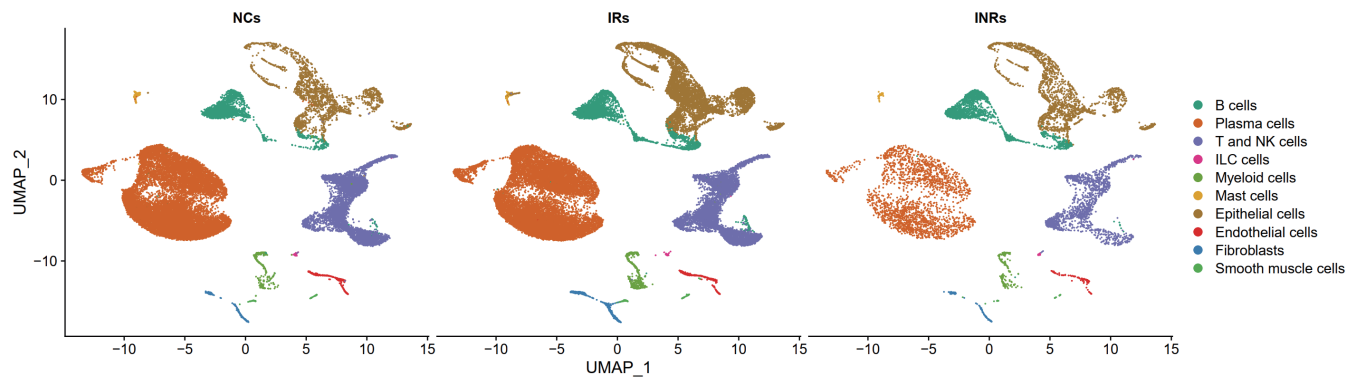

B

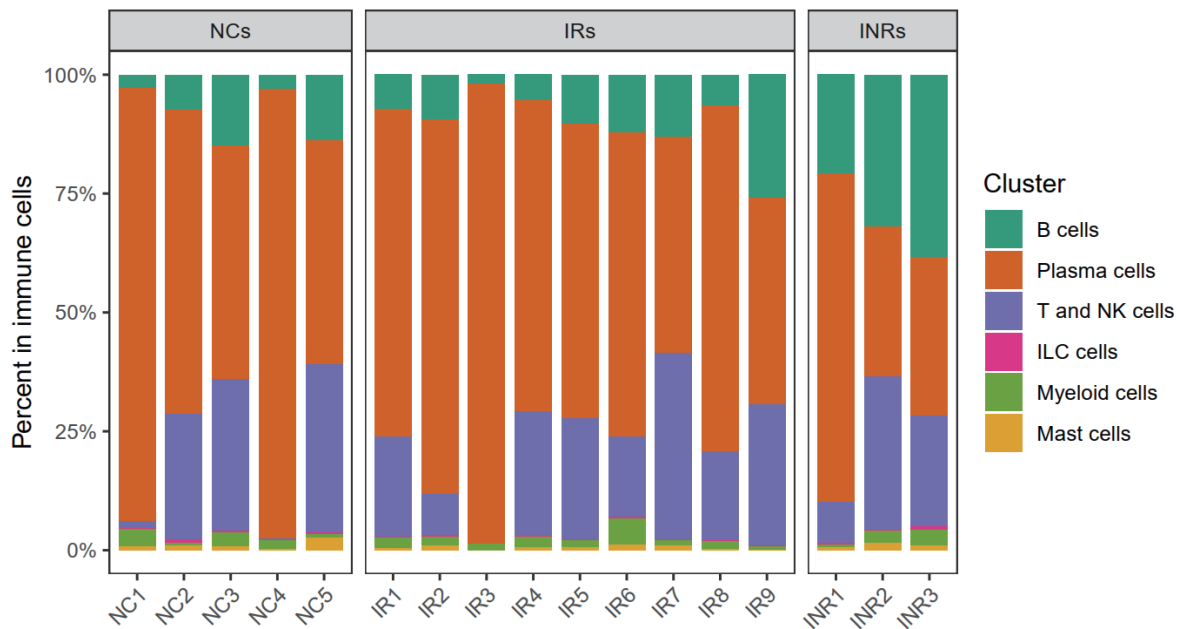

Supplement: Supplementary file 2 — Supporting Information [file CTM2-14-e1699-s001.pdf]

A

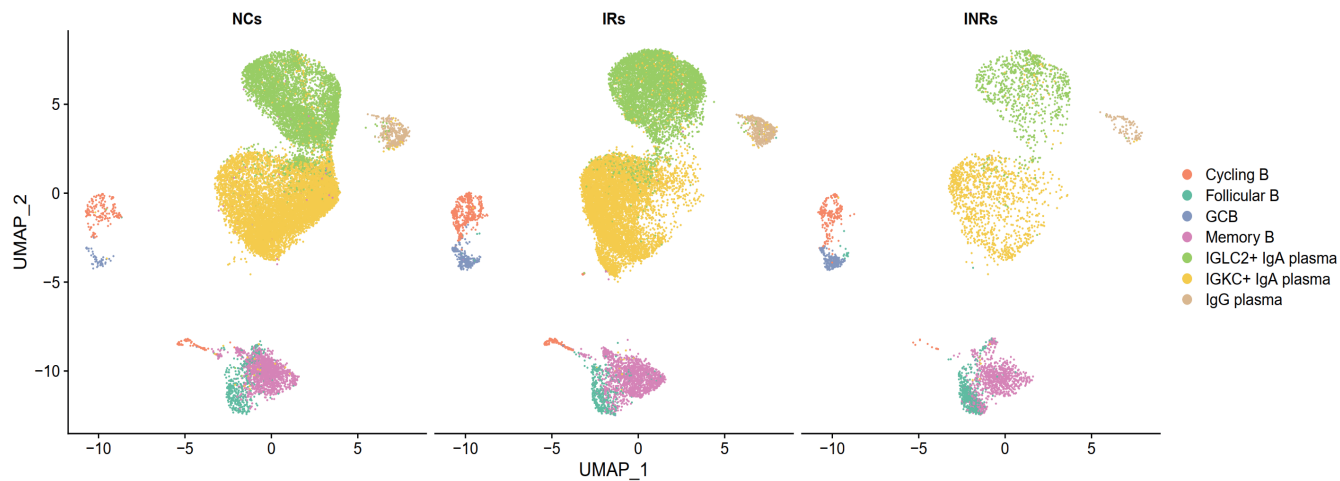

B

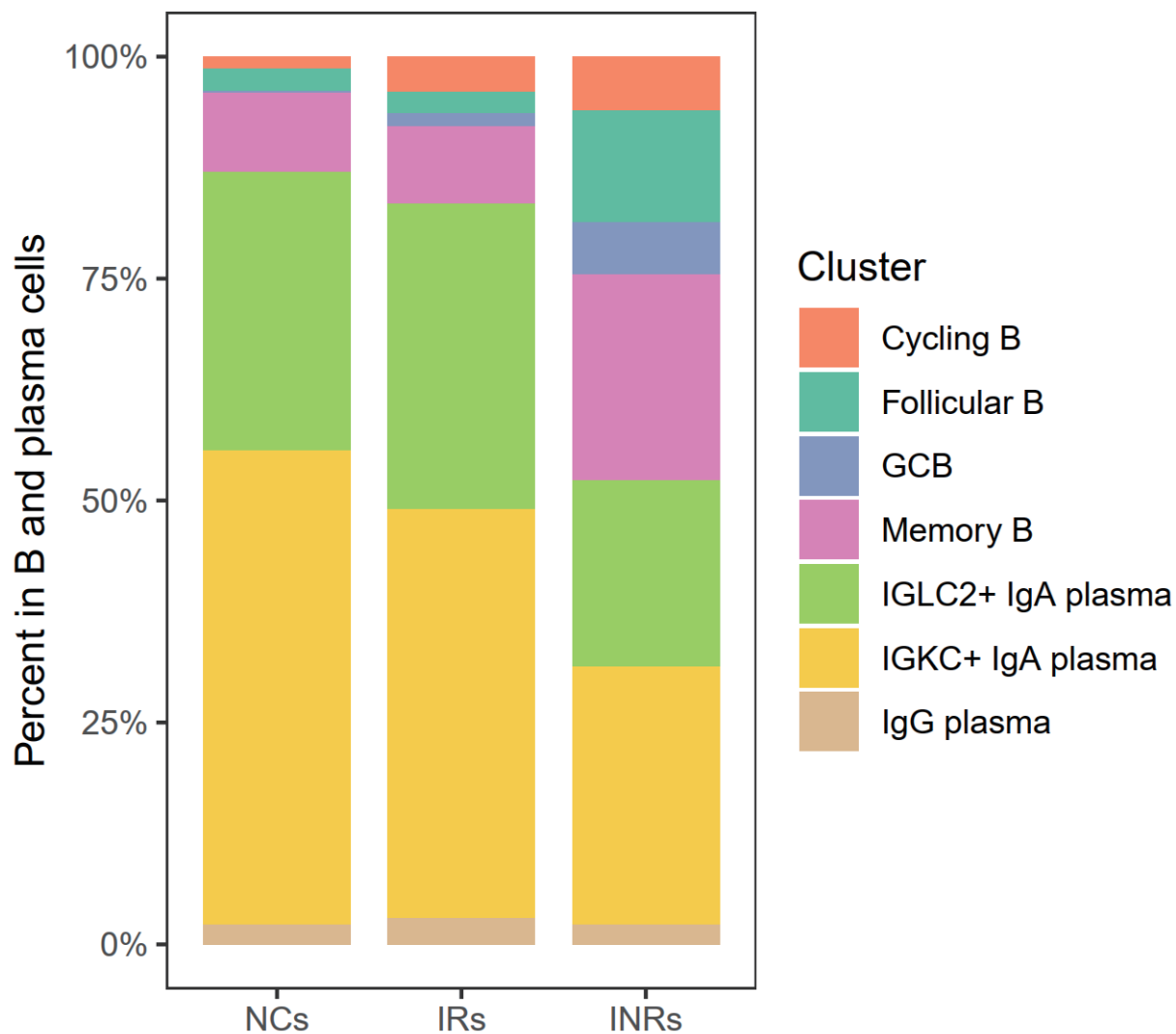

Supplement: Supplementary file 3 — Supporting Information [file CTM2-14-e1699-s005.pdf]

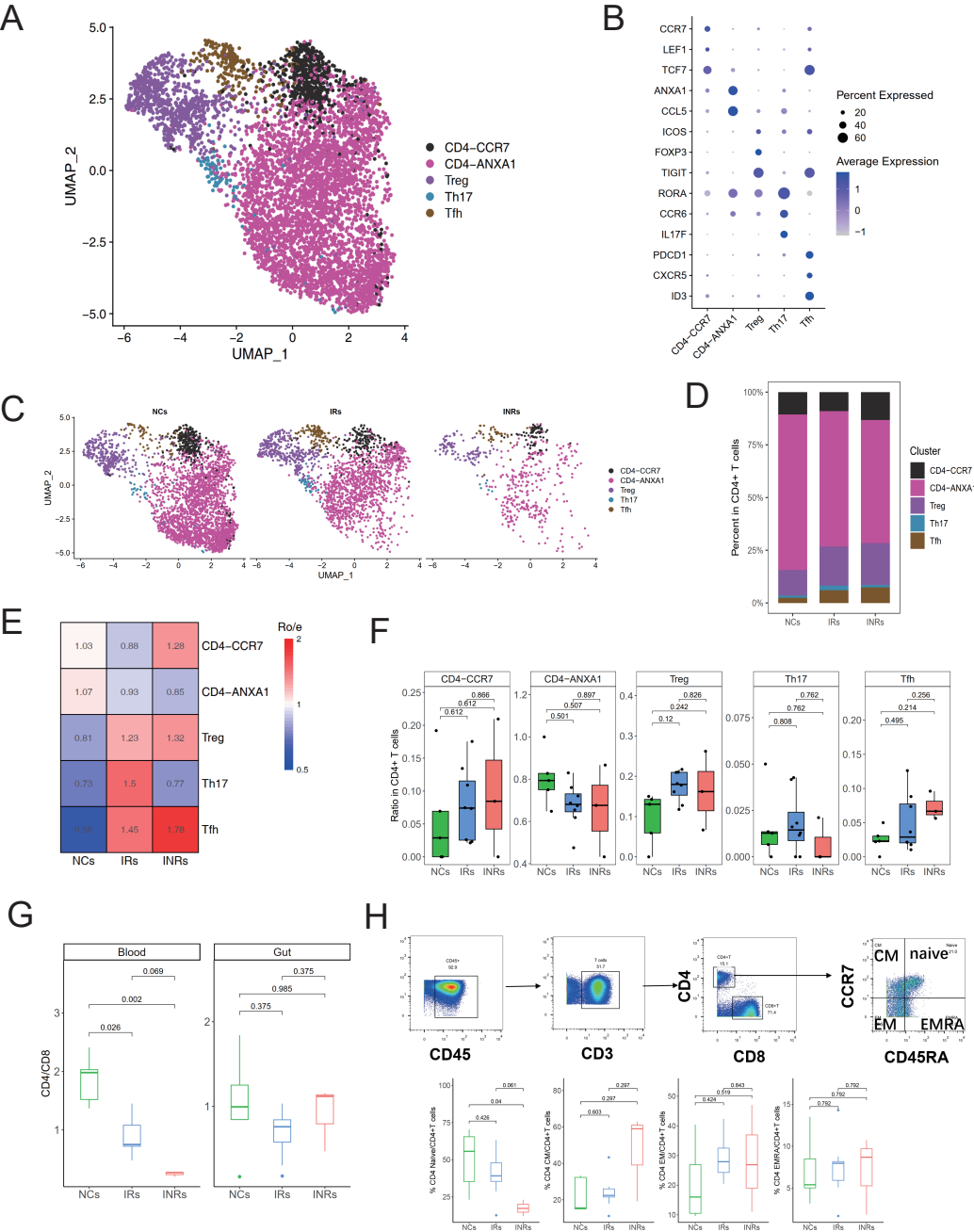

Supplement: Supplementary file 4 — Supporting Information [file CTM2-14-e1699-s002.pdf]

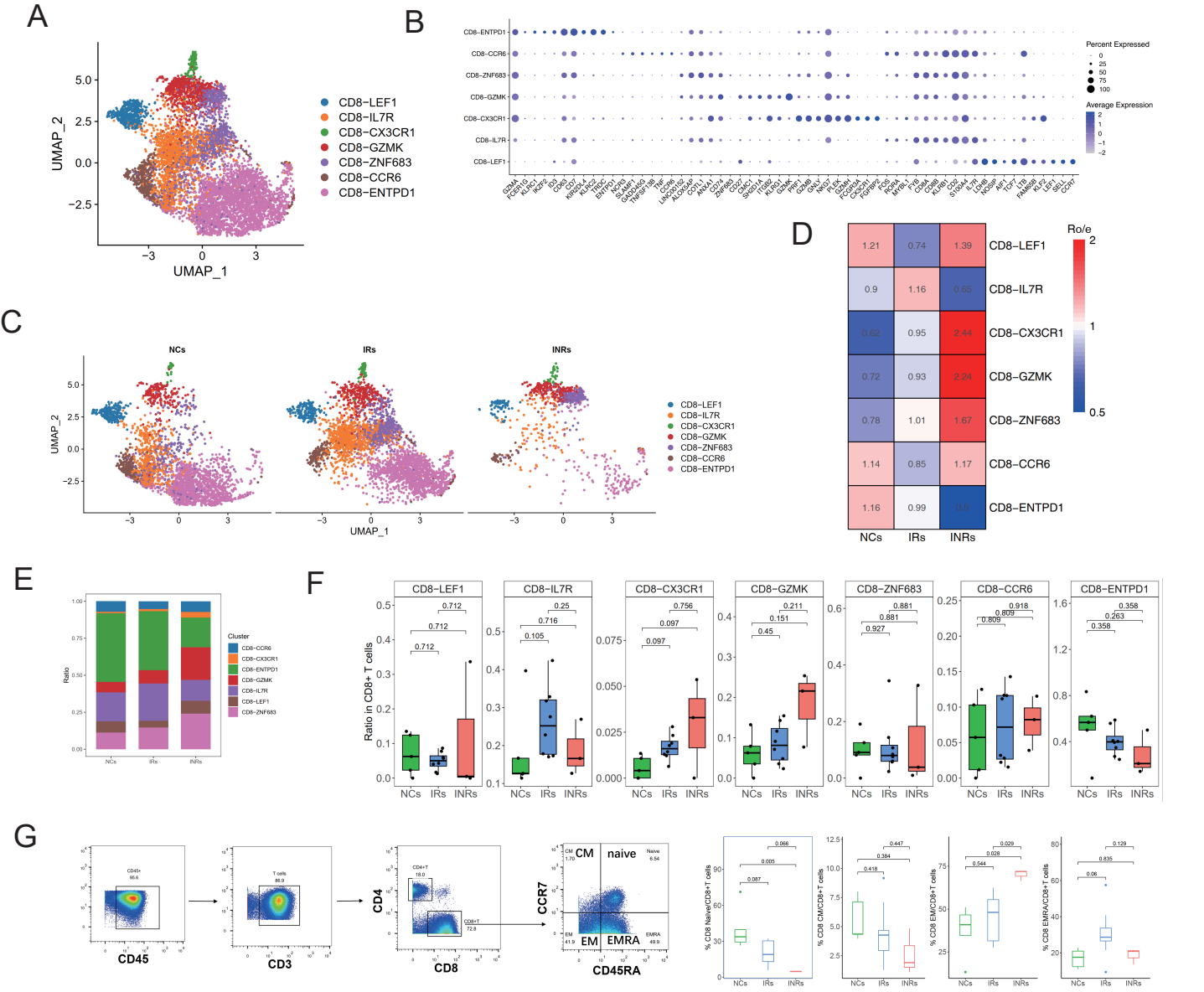

Supplement: Supplementary file 5 — Supporting Information [file CTM2-14-e1699-s003.pdf]

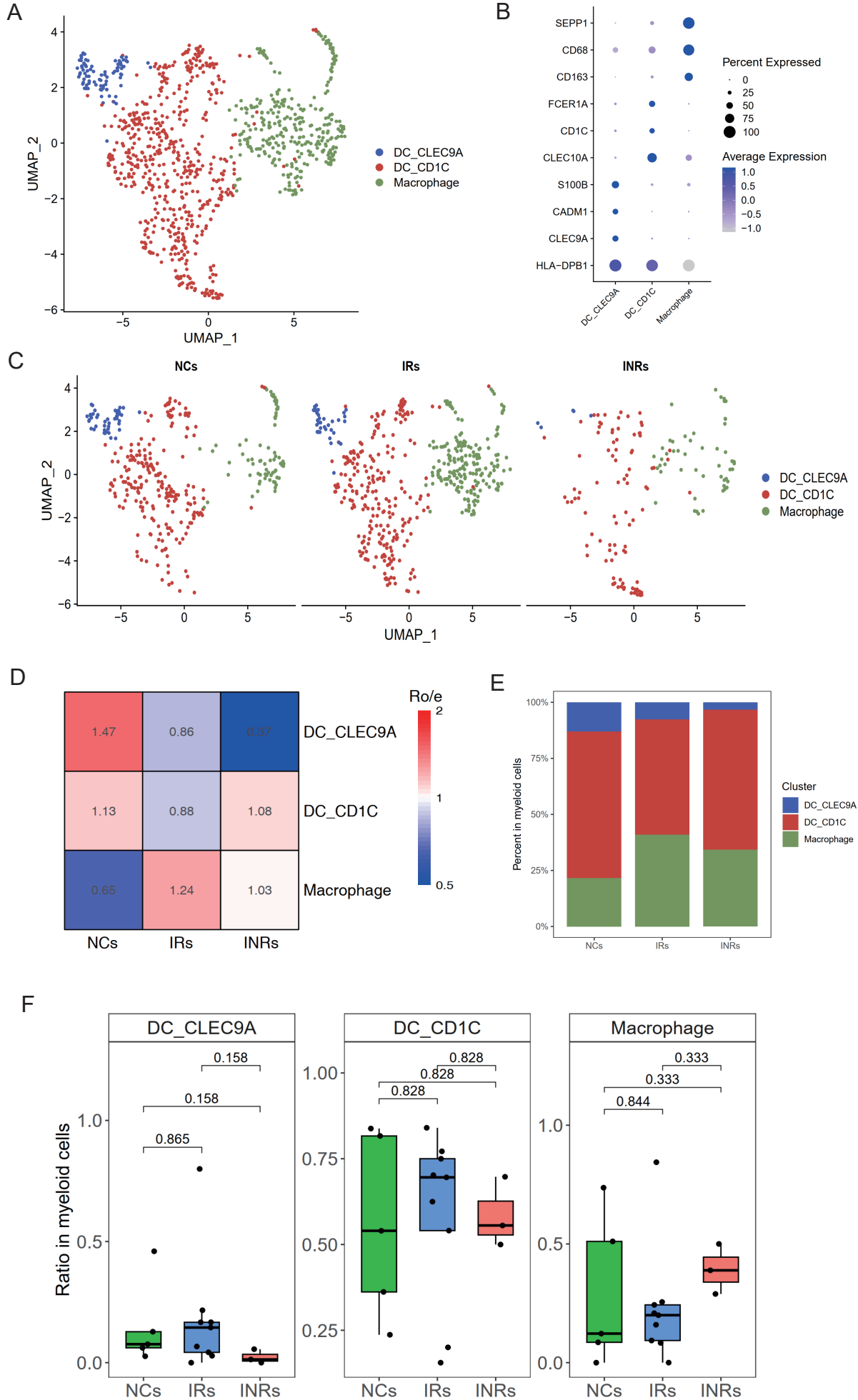

Supplement: Supplementary file 6 — Supporting Information [file CTM2-14-e1699-s004.pdf]
